# Early psychiatric referral after attempted suicide helps prevent suicide reattempts: A longitudinal national cohort study in South Korea

**DOI:** 10.3389/fpsyt.2022.607892

**Published:** 2022-09-06

**Authors:** Hyewon Kim, Yuwon Kim, Myung-Hee Shin, Yoo-Jung Park, Hyung-Eun Park, Maurizio Fava, David Mischoulon, Mi Jin Park, Eun Ji Kim, Hong Jin Jeon

**Affiliations:** ^1^Department of Psychiatry, Hanyang University Hospital, Seoul, South Korea; ^2^Department of Data Science, Evidnet, Seongnam, South Korea; ^3^Department of Social and Preventive Medicine, Sungkyunkwan University School of Medicine, Seoul, South Korea; ^4^Pfizer Pharmaceuticals Korea Ltd., Seoul, South Korea; ^5^Depression Clinical and Research Program, Massachusetts General Hospital, Harvard Medical School, Boston, MA, United States; ^6^Department of Psychiatry, College of Medicine, Seoul St. Mary’s Hospital, The Catholic University of Korea, Seoul, South Korea; ^7^Department of Psychiatry, Depression Center, Samsung Medical Center, Sungkyunkwan University School of Medicine, Seoul, South Korea; ^8^Department of Health Sciences and Technology, Samsung Advanced Institute for Health Sciences and Technology (SAIHST), Sungkyunkwan University, Seoul, South Korea; ^9^Department of Medical Device Management and Research, Samsung Advanced Institute for Health Sciences and Technology (SAIHST), Sungkyunkwan University, Seoul, South Korea; ^10^Department of Clinical Research Design and Evaluation, Samsung Advanced Institute for Health Sciences and Technology (SAIHST), Sungkyunkwan University, Seoul, South Korea

**Keywords:** suicide attempt, suicide reattempt, psychiatric service use, psychiatric referral, suicide prevention

## Abstract

**Introduction:**

Although people who attempted suicide tend to repeat suicide attempts, there is a lack of evidence on the association between psychiatric service factors and suicide reattempt among them.

**Methods:**

We used a nationwide, population-based medical record database of South Korea to investigate the use of psychiatric services before and after the index suicide attempt and the association between psychiatric service factors after the index suicide attempt with the risk of suicide reattempt.

**Results:**

Among 5,874 people who had attempted suicide, the all-cause mortality within 3 months after the suicide attempt was 11.6%. Among all subjects who attempted suicide, 30.6% of them had used psychiatric services within 6 months before the suicide attempt; 43.7% of them had used psychiatric services within 3 months after the suicide attempt. Among individuals who had visited clinics following attempted suicide, the cumulative incidence of suicide reattempt over a mean follow-up period of 5.1 years was 3.4%. About half of suicide reattempts occurred within 1 year after the index suicide attempt. Referral to psychiatric services within 7 days was associated with a decreased risk of suicide reattempt (adjusted hazard ratio, 0.51; 95% confidence intervals, 0.29–0.89).

**Conclusion:**

An early psychiatric referral within 1 week after a suicide attempt was associated with a decreased risk of suicide reattempt.

## Introduction

Suicide is a serious public health problem and a major cause of death worldwide (1). Globally, approximately 700,000 people die by suicide every year (2), and there are more than 20 failed suicide attempts for each suicide (3). The incidence of suicide varies across countries, and it is relatively high in South Korea with 26.9 per 100,000 people (4). Although no single risk factor is a strong predictor of suicide (5), previous studies identified risk factors associated with suicidal behaviors as genetic loading (6, 7), brain dysfunction (8, 9), psychopathologies (10, 11), emotional states, such as aggression and impulsivity (7, 12), comorbid physical diseases (13, 14), the use of hypnotics or illicit substances (7), and personal experiences, such as childhood trauma (6, 15).

There are two approaches to the management of suicidal people: suicide-specific treatments and treating the underlying psychiatric disorder (5). Suicide-specific treatments include long-term psychosocial interventions, brief psychosocial interventions, and pharmacological interventions; studies showed the antisuicidal effect of cognitive behavioral therapy (16), dialectical behavior therapy (17), caring contacts (18), and medications such as lithium (19, 20) and ketamine (21, 22). About 60–98% of suicide deaths are associated with primary psychiatric disorders (23). Depression, substance use disorders, schizophrenia, and personality disorders are the main psychiatric disorders that are associated with an increased risk of suicide (24). Moreover, a reduction in the professional care of patients with mental illness showed a strong association with suicide (25), whereas studies showed that the implementation of mental health services can reduce the risk of suicide (26, 27), suggesting the provision of mental health services to be an important aspect to prevent suicide.

A previous suicide attempt is one of the robust predictors of future suicide; about 30% of people who attempt suicide are known to repeat suicide attempts (28). Therefore, people with a history of suicide attempts were included in a high-risk group for suicide and were targeted for secondary prevention of suicide. Although numerous studies discovered the predictors for suicidal behaviors as previously mentioned, clinical predictors for suicide reattempt among subjects who attempted suicide are relatively poorly understood. A community-based study reported age ≤ 25 years, a higher family income, having any psychiatric disorder, poor education, stressful life events, alcohol abuse, and smoking to be associated with suicide reattempts (29). A study conducted on a single study site reported cluster B personality disorder, good treatment compliance, and repeated suicide attempts to be associated with an increased risk of suicide reattempt among subjects who attempted suicide (30). In an observational study, scheduling a single appointment within 7 days after discharge from the emergency room following a suicide attempt was found to lower the risk of suicide reattempt (31). Repeated follow-up among the clinical population was found to reduce the risk of suicide reattempt (32, 33).

The aim of this study was to expand the evidence by investigating the association between psychiatric service use factors with suicide reattempt among subjects who attempted suicide. We hypothesized that (1) the use of psychiatric services after a suicide attempt would prevent suicide reattempt and (2) the timing and frequency of referral to psychiatry after a suicide attempt would be associated with the risk of suicide reattempt.

## Materials and methods

### Data source

This study was based on the National Health Insurance Sharing Service (NHISS) database of the National Health Insurance Service (NHIS) of South Korea (34, 35). NHIS is a public institution responsible for operationalizing mandatory universal health insurance; approximately 97% of the total population in South Korea is enrolled in this service, while the remaining 3% is covered by the Medical Aid Program. The NHISS database contains medical services claim data (such as information about admission, emergency room visits, ambulatory care visits, and pharmaceutical services) and data on health screening programs. The NHISS data are anonymized to protect the privacy of individuals. The study protocol was approved by the Institutional Review Board of the Samsung Medical Center (No. 2019-03-136).

### Population

We used NHISS data for the period from 1 January 2002 to 31 December 2018. We selected people who attempted suicide from 1 January 2003 to 31 December 2017 as subjects to rule out any previous suicide attempt in at least 1 year immediately preceding the index suicide attempt and to ensure a follow-up period of at least 1 year after the index suicide attempt.

People with intentional self-harm codes were identified as subjects based on the International Statistical Classification of Disease and Related Health Problems, 10th revision (ICD-10). We included people aged ≥ 18 years with X60–84 of the ICD-10 codes in the analyses.

### Psychiatric service use before the index suicide attempt

We identified psychiatric service use of subjects before the index suicide attempt based on psychiatric outpatient visits, hospitalization in psychiatric wards, or emergency room care within 6 months before the index suicide attempt; psychiatric consultations made during hospitalization in other departments were also included based on the codes of individual psychotherapy. In addition, subjects’ psychiatric diagnosis and the use of psychiatric medications including antidepressants, antipsychotics, benzodiazepines, stimulants, mood stabilizers, and zolpidem were identified.

### Deaths after the index suicide attempt

We identified all-cause deaths within the 3 months after the index suicide attempt. Considering the individual differences in the time leading to death, the period was categorized as 7 days, 8–14 days, 15–28 days, and 29–90 days.

### Use of psychiatric services after the index suicide attempt

We operationally set 3 months to assess the psychiatric service use related to the index suicide attempt, considering the maximum prescribing period for psychiatric medications in South Korea. During this period, psychiatric outpatient visits, hospitalizations in psychiatric wards, emergency room care, and psychiatric consultations were identified. In addition, the timing of the first visit to the psychiatrist after the index suicide attempt, the frequency of psychiatric care visits, and the use of psychiatric medications during this period were identified.

### Suicide reattempts

The primary outcome was suicide reattempts occurring from 3 months after the index suicide attempt. The results obtained within the 3 months immediately after the index suicide attempt were not included in the main outcome analysis; this was because, during this period, the suicide attempt codes could have been used not only for the actual suicide reattempt but also for medical care related to the index suicide attempt. Accordingly, we investigated the occurrence of suicide reattempts in people who had survived the attempt and had the code of suicide attempt from 3 months after the index suicide attempt.

### Statistical analysis

We presented the distribution of demographic and socioeconomic characteristics, psychiatric characteristics, survival outcomes, and psychiatric service use factors after the index suicide attempts as numbers and percentages. The incidence of suicide reattempt was calculated, and multivariate regression analyses were performed to calculate the hazard ratios (HRs) for potential risk factors associated with suicide reattempt after adjusting for sex, age, disability severity, psychiatric diagnosis, referral to a psychiatrist within 3 months after the index suicide attempt, and psychiatric medication use within 3 months after the index suicide attempt. All statistical analyses were performed using SAS software version 9.4 (SAS Institute Inc., Cary, NC, United States).

## Results

### Demographic and socioeconomic characteristics of subjects who attempted suicide

Table 1 and Supplementary Table 1 show the demographic and socioeconomic characteristics of subjects who attempted index suicide (*n* = 5,874); of these, 51.5% were men, and individuals in the age group of 40–49 years constituted the largest subgroup (19.1%). We categorized the study population into income classes based on the payment of health insurance (In South Korea, the payment of health insurance is determined by income level). Individuals belonging to the upper class accounted for the highest proportion of subjects who attempted index suicide (37.4%), followed by the middle class (35.2%). Approximately 1.3% of the subjects who attempted index suicide had a psychiatric disability, while 12.7% had other disabilities.

**TABLE 1:** Demographic and socioeconomic characteristics of the study population.

	Total subjects who attempted suicide (*n* = 5,874)	Psychiatric service use before the index suicide attempt^a^		
		Yes (*n* = 1,798)	No (*n* = 4,076)	*P-value*
	*N* (%)	*N* (%)	*N* (%)	
Sex				<0.001
Men	3,027 (51.5)	824 (45.8)	2,203 (54.1)	
Women	2,847 (48.5)	974 (54.2)	1,873 (46.0)	
Age (years)				0.037
10–19	341 (5.8)	93 (5.2)	248 (6.1)	
20–29	711 (12.1)	215 (12.0)	496 (12.2)	
30–39	910 (15.5)	301 (16.7)	609 (14.9)	
40–49	1,121 (19.1)	357 (19.9)	764 (18.7)	
50–59	1,081 (18.4)	338 (18.8)	743 (18.2)	
60–69	658 (11.2)	186 (10.3)	472 (11.6)	
70–79	631 (10.7)	204 (11.4)	427 (10.5)	
≥80	421 (7.2)	104 (5.8)	317 (7.8)	
Income class^b^				<0.001
Lower	1,407 (27.4)	381 (26.3)	1,026 (27.8)	
Middle	1,812 (35.2)	469 (32.3)	1,343 (36.4)	
Upper	1,924 (37.4)	601 (41.4)	1,323 (35.8)	
Disability type				<0.001
None	5,055 (86.1)	1,482 (82.4)	3,573 (87.7)	
Psychiatric disability	75 (1.3)	75 (4.2)	0 (0)	
Other disabilities^c^	744 (12.7)	241 (13.4)	503 (12.3)	
Disability severity				<0.001
No disability	5,055 (86.1)	1,482 (82.4)	3,573 (87.7)	
Mild	460 (7.8)	147 (8.2)	313 (7.7)	
Severe	359 (6.1)	169 (9.4)	190 (4.7)	

^a^Psychiatric service use within 6 months before the index suicide attempt.

^b^Income class stratified according to the level of health insurance payment; lower class.

^c^Includes physical disability, disability caused by brain lesions, visual disability, hearing impairment, speech impairment, and intellectual disability.

Among all the subjects who attempted suicide, 30.6% had visited a psychiatry clinic within 6 months before the index suicide attempt. On stratifying the study population according to psychiatric service use before the index suicide attempt, there were significant differences in the proportion of psychiatric service use before the index suicide attempt by sex, age, residential area, household income, type of disability, and severity of the disability. Men (27.2%) and those above 80 years (24.9%) showed a prominently lower proportion of psychiatric service use before the index suicide attempt.

### Psychiatric characteristics of the study population

We investigated the psychiatric characteristics of the study population before the index suicide attempt. The diagnoses of adjustment disorder, depressive disorder, schizophrenia spectrum disorder, bipolar disorder, or other psychiatric disorders were included in the analysis if identified as a primary diagnosis made by a psychiatrist. Insomnia was included in the analysis if it was a primary or secondary diagnosis made by a doctor working in any department. About 39.9% of the subjects were diagnosed with insomnia. Based on the primary diagnosis by a psychiatrist, depressive disorders were the most prevalent psychiatric disorder among subjects who attempted suicide (19.4%), while insomnia accounted for 5.3%; 49.6% of subjects had no psychiatric diagnosis.

Within 6 months before the index suicide attempt, 44.5, 22.8, and 19.8% of the subjects had been prescribed benzodiazepines, antidepressants, and zolpidem, respectively. During this period, 27.4% had visited the psychiatric outpatient clinic and 6.4% had been admitted to the psychiatric wards. In the course of the entire study period before the index suicide attempt, 13.7% had a history of admission to a psychiatric ward (Table 2).

**TABLE 2:** Psychiatric characteristics of subjects who attempted suicide before the index suicide attempt.

	Subjects who attempted suicide (*N* = 5,874)	
	N	%
Psychiatric diagnosis^a^		
Adjustment disorder	102	1.7
Depressive disorder	1,137	19.4
Schizophrenia spectrum disorders	209	3.6
Bipolar disorder	244	4.2
Insomnia	2,343	39.9
Other psychiatric disorders	1,355	23.1
No psychiatric diagnosis	2,912	49.6
Psychiatric medication use^b^		
Antidepressants	1,340	22.8
Antipsychotics	836	14.2
Benzodiazepines	2,616	44.5
Stimulants	125	2.1
Mood stabilizers	423	7.2
Zolpidem	1,161	19.8
Psychiatric outpatient visits	1,609	27.4
within 6 months before the index		
suicide attempt		
Admission to the psychiatric	375	6.4
ward within 6 months before the		
index suicide attempt		
Admission to the psychiatric	806	13.7
ward prior to the index suicide		
attempt during the study period		

^a^Insomnia was diagnosed by doctors of any department, while the other conditions were diagnosed by a psychiatrist (as the main diagnosis) during the study period before the index suicide attempt.

^b^The use of psychiatric medication in the 6-month period immediately preceding the index suicide attempt.

### Methods used for the index suicide attempt

Approximately 68.9% of subjects who attempted suicide tried to poison themselves. Hanging, strangulation, and suffocation accounted for 12.0%, and the use of sharp or blunt objects accounted for 10.9% of suicide attempts (Supplementary Table 2).

### Deaths and psychiatric service use within 3 months after an index suicide attempt

Among subjects who attempted index suicide, 88.4% survived 3 months after the index suicide attempt, while 8.0% died within 7 days. Approximately 43.7% of subjects had used psychiatric services within 3 months after the index suicide attempt. After the index suicide attempt, 32.9% had visited their psychiatric service within 7 days, 7.7% within 8–28 days, and 3.1% within 29 days–3 months (Table 3).

**TABLE 3:** Deaths and psychiatric service use of subjects who attempted index suicide within 3 months after the index suicide attempt.

	Subjects who attempted suicide (*N* = 5,874)	
	N	%
Survival outcome		
Alive	5,193	88.4
Died in 7 days	470	8.0
Died in 8–14 days	48	0.8
Died in 15–28 days	51	0.9
Died in 29–90 days	112	1.9
Referral to psychiatrist		
None	3,309	56.3
Within 7 days	1,935	32.9
Within 8–28 days	451	7.7
Within 29 days–3 months	179	3.1
Frequency of visits to the psychiatrist		
0	3,309	56.3
1–2	880	15.0
≥3	845	14.4
Admission to psychiatry	840	14.3
Psychiatric medication use		
Antidepressants	1,440	24.5
Antipsychotics	1,041	17.7
Benzod iazepines	2,234	38.0
Stimulants	78	1.3
Mood stabilizers	491	8.4
Zolpidem	871	14.8

### Incidence of suicide reattempt after the index suicide attempt

**FIGURE 1:**
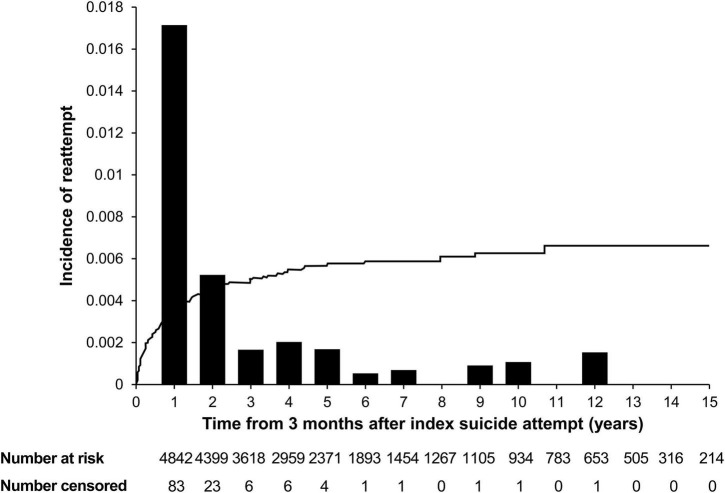
Incidence of suicide reattempt after an index suicide attempt. Incidence of suicide reattempt among individuals who visited clinics with attempted suicide from 3 months after the index suicide attempt. The line represents the cumulative incidence of suicide reattempt after the index suicide attempt, while the bars represent the yearly incidence of suicide reattempt after the index suicide attempt. Yearly incidence: Year 1 = 1.7%, Year 2 = 0.5%, Year 3 = 0.2%, Year 4 = 0.2%, Year 5 = 0.2%, Year 6 = 0.1%, Year 7 = 0.1%, Year 8 = 0%, Year 9 = 0.1%, Year 10 = 0.1%, Year 11 = 0%, Year 12 = 0.2%, Year 13 = 0%, Year 14 = 0%, and Year 15 = 0%.

We investigated the incidence of suicide reattempts from 3 months after the index suicide attempt. The incidence of suicide reattempt in the entire study population over a mean follow-up period of 5.1 years was 3.36% (95% CI: 2.74–4.11). The yearly incidence was the highest in the first year from 3 months after the index suicide attempt (year 1 = 1.7%) (Figure 1).

**FIGURE 2:**
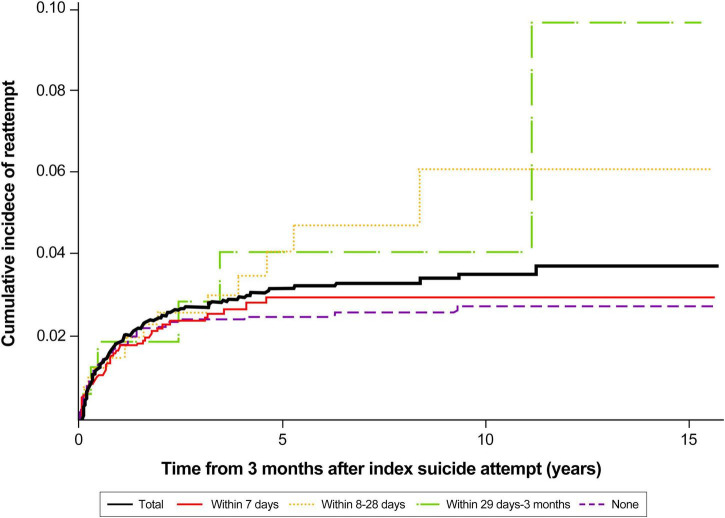
Cumulative incidence of suicide reattempt after index suicide attempt according to the timing of referral to psychiatry.

Regarding referral to a psychiatrist after the index suicide attempt, the incidence of suicide reattempt was 2.82% (95% CI: 2.16–3.69) among individuals who were not referred within 3 months, 3.04% (95% CI: 2.25–4.12) among those who were referred within 7 days, 6.08% (95% CI: 3.29–11.08) among those who were referred within 8–28 days, and 9.73% (95% CI: 2.98–29.30) among those who were referred within 29 days–3 months (Figure 2).

### Adjusted hazard ratios of potential risk factors for suicide reattempts

We investigated the potential risk factors for suicide reattempt in the study population and calculated the corresponding HRs. Mild disability was associated with a significantly higher risk of suicide reattempt compared to the absence of any disability [adjusted hazard ratio (aHR) 2.09, 95% confidence interval (CI): 1.21–3.62, *p* = 0.008]. Adjustment disorder, depressive disorder, schizophrenia spectrum disorder, bipolar disorder, and insomnia showed no significant association with suicide reattempt. Referral to a psychiatrist within 7 days from the index suicide attempt was associated with a reduced risk of suicide reattempt compared to the absence of psychiatric referral (aHR 0.51, 95% CI: 0.29–0.89, *p* = 0.018). The use of antidepressants was associated with an increased risk of suicide reattempt (aHR 1.73, 95% CI: 1.08–2.77, *p* = 0.022), while the use of zolpidem was associated with a decreased risk of suicide reattempt (aHR 0.58, 95% CI: 0.34–0.99, *p* = 0.045) compared to the non-use of psychiatric medications (Table 4).

**TABLE 4:** Adjusted hazard ratios of potential risk factors for suicide reattempt among subjects who attempted suicide had not reattempted suicide or died within the 3-month period after the index suicide attempt (*n* = 4,842)^a^.

	Hazard ratio (95% confidence intervals)	*P-value*
Female sex	0.86 (0.59–1.23)	0.401
Age (years)		
≤18	1 (Ref.)	
19–34	0.78 (0.32–1.92)	0.589
35–49	0.81 (0.33–1.94)	0.630
50–64	0.88 (0.36–2.16)	0.786
≥65	0.57 (0.22–1.48)	0.246
Disability severity		
No disability	1 (Ref.)	
Mild	2.09 (1.21–3.62)	0.008
Severe	1.70 (0.93–3.12)	0.086
Psychiatric diagnosis^b^		
Adjustment disorder	0.67 (0.25–1.84)	0.441
Depressive disorder	1.07 (0.70–1.65)	0.755
Schizophrenia spectrum disorder	1.15 (0.61–2.15)	0.667
Bipolar disorder	1.18 (0.65–2.15)	0.584
Insomnia	1.49 (0.96–2.31)	0.078
Other psychiatric disorders	1.50 (1.01–2.23)	0.044
Referral to a psychiatrist within 3 months after the index suicide attempt		
None	1 (Ref.)	
Within 7 days	0.51 (0.29–0.89)	0.018
Within 8–28 days	0.69 (0.34–1.39)	0.299
Within 29 days–3 months	0.75 (0.30–1.88)	0.543
Psychiatric medication use within 3 months after the index suicide attempt		
No psychiatric medication	1 (Ref.)	
Antidepressants	1.73 (1.08–2.77)	0.022
Antipsychotics	1.57 (0.92–2.69)	0.098
Benzodiazepines	1.02 (0.66–1.60)	0.923
Stimulants	0.54 (0.07–3.85)	0.535
Mood stabilizers	1.27 (0.71–2.26)	0.414
Zolpidem	0.58 (0.34–0.99)	0.045

^a^Adjusted for sex, age, disability severity, psychiatric diagnosis, referral to a psychiatrist within 3 months after the index suicide attempt, and psychiatric medication use within 3 months after the index suicide attempt.

^b^Insomnia was diagnosed by doctors of any department, while the other conditions were diagnosed by a psychiatrist (as the main diagnosis) during the study period.

On multivariate regression analysis after including the frequency of visits to a psychiatrist during the 3 months instead of referral to a psychiatrist for 3 months, one or two visits after the index suicide attempt were associated with a lower risk of suicide reattempt compared to no visit (aHR 0.25, 95% CI: 0.10–0.59, *p* = 0.002) (Supplementary Table 3).

## Discussion

In this study, we used national claims data to investigate the psychiatric service use factors of subjects who attempted suicide before and after their initial suicide attempt and the association between psychiatric service use after an index suicide attempt and the risk of suicide reattempt. We found that approximately 30% of the subjects who attempted suicide had visited psychiatry within 6 months before the index suicide attempt. About 8 and 12% of subjects who attempted suicide died within 7 days and 3 months after the index suicide attempt, respectively; therefore, the estimated mortality rate for index suicide attempts was 8–12%. Mild disability and the use of antidepressants were associated with an increased risk of suicide reattempt, while the use of zolpidem was associated with a decreased risk of suicide reattempt. Referral to psychiatry within 7 days after the index suicide attempt was associated with a significantly lower risk of suicide reattempt.

While most suicidal behaviors are known to be related to psychiatric diseases, only 30% of the study population had visited psychiatry clinics prior to their index suicide attempt. This finding indicates the undertreatment of the psychiatric problems of subjects who attempted suicide. This phenomenon is likely attributable to access to care. Although the access to care in South Korea could be evaluated as high, considering the high level of healthcare use, low avoidable mortality, and low infant mortality (36), it may differ by individual factors. Therefore, it is necessary to identify the population with low access to care and to prevent any delay in psychiatric intervention for them to prevent suicide reattempt. In addition, the stigma associated with mental illnesses could affect the undertreatment (37); people with suicidal ideation tend to hide their suicidality and refrain from seeking professional help (38–40). Even after it became clear that these individuals were at high risk of suicide after the failed suicide attempt, referrals to psychiatry increased to only 44%. In this study, there were significant differences in the proportion of psychiatric service use before the index suicide attempt by demographic and socioeconomic characteristics, and men and those above 80 years showed further lower use of psychiatric service use before the index suicide attempt, suggesting that they could be high-risk populations for the undertreatment. In a previous study, age, presence of psychotic disorder, and absence of substance use disorder were predictors of referral to psychiatric consultation after the suicide attempt, while the hospital where the subjects who attempted suicide were treated most strongly affected the referral to psychiatry (41). Considering these, additional efforts are needed to ensure the subsequent psychiatric intervention among subjects who attempted suicide.

Referral to psychiatry within 7 days after the index suicide attempt reduced the risk of suicide reattempt by half compared to those who did not visit psychiatry; this suggests the importance of rapid psychiatric intervention after the suicide attempt for secondary prevention of suicide attempts. According to a previous study of subjects who attempted suicide, approximately 49% of subjects required urgent psychiatric care during the follow-up period (42). Indeed, even among subjects who attempted suicide with no mental illness, immediate and brief intervention after suicide attempts resulted in lowering the subsequent suicide rate (43). Early psychiatric intervention can help prevent further suicide attempts by assisting the individual in dealing with suicide-associated stigma and stressful life events in addition to facilitating treatment of mental illnesses. A qualitative study found that the stigma associated with mental illness contributes to suicidality; moreover, suicide survivors experience additional suicide-related stigma (37). Similarly, another study revealed an association between the anticipated stigma of suicide and suicidality; this suggests that the anticipated suicide-related stigma and secrecy can aggravate suicidality in subjects who attempted suicide (44). Suicide-related stigma can lead to a feeling of loneliness or hopelessness, which are precursors of suicidality (37). Thus, we assume that timely psychiatric intervention in the early stages of suicidal behavior may help prevent suicide reattempts.

The use of antidepressants was associated with an increased risk of suicide reattempts, and the use of zolpidem was associated with a decreased risk of them. However, these results should be interpreted with caution as our study is based on claims data; data about clinical variables (such as the severity of depression) were not adequately captured in the database. Regarding antidepressant use, those who used antidepressants might have greater severity of depression or a recurrent course than those who did not use antidepressants, which may affect the increased risk of suicide reattempt. Indeed, there is no clear consensus on the association between the use of antidepressants and suicide risk. After the Food and Drug Administration had issued a black-box warning reflecting reports of an increased risk of suicidal behavior during treatment of young individuals with antidepressants (45, 46), the prescription of antidepressants and the frequency of diagnosis of depression showed a decline, and these resulted in a concomitant increase in the incidence of psychotropic drug poisoning, which is considered a proxy for suicide attempts (47). Similarly, regarding the decreased risk of suicide reattempt among zolpidem users, although this finding is consistent with that of our previous study (48), there also have been several studies reporting the opposite results (49–51). The reason for these opposite results is assumed that various confounders related to the use of zolpidem can affect suicidal behavior and methodologic differences in consideration of this might differ between studies. That is, in addition to the single factor of “use of zolpidem,” various clinical factors in the individual might affect suicidal behaviors, such as degree of insomnia, other psychiatric medications, and comorbid mental disorders.

This study has strength, in that it employed real-world data based on a national sample to characterize the use of psychiatric services by subjects who attempted suicide and investigated the long-term effects of potential risk factors for suicide reattempt. However, some limitations of this study should be considered while interpreting the results. First, we used intentional self-harm codes as per ICD-10 to define subjects who attempted suicide. Self-harm by concept includes suicide attempts; however, it is possible that people with non-suicidal self-harm were also included in this study. Second, we operationally set a 3-month period after the index suicide attempt for the occurrence of deaths and psychiatric service use after the index suicide attempt and followed up suicide reattempts after this period. However, it is highly possible that suicide reattempt occurs within 3 months after the index suicide attempt. A previous study found that about 50% of suicide reattempts occur within the first 6 months after the index suicide attempt (30). Similarly, delayed management following the index suicide attempt might have been included in the analysis for suicide reattempt.

## Conclusion

This retrospective cohort study using national claims data discovered that early psychiatric referral of subjects who attempted suicide, especially within 1 week after the initial attempt, can reduce the risk of suicide reattempt. Individuals who attempt suicide tend to repeat suicide attempts; in the process, their suicidal capability increases with a consequent increase in the probability of death (52). Physicians should be aware of this phenomenon and identify individuals undergoing this process; immediate referrals of these individuals to psychiatry may help stop this devastating process.

## Data availability statement

Publicly available datasets were analyzed in this study. This data can be found here: https://nhiss.nhis.or.kr/ (Research management number: NHIS-2019-1-520).

## Ethics statement

The studies involving human participants were reviewed and approved by the Institutional Review Board of the Samsung Medical Center. Written informed consent for participation was not required for this study in accordance with the national legislation and the institutional requirements.

## Author contributions

HK contributed to the conceptualization, data curation, investigation, methodology, and writing the original draft of the manuscript. YK and M-HS contributed to the data analysis. H-EP and Y-JP contributed to the conceptualization and project administration. HJJ contributed to the conceptualization, project administration, and supervision. All authors contributed to the writing and editing the manuscript.
