# Supplementary material for: Early psychiatric referral after attempted suicide helps prevent suicide reattempts: A longitudinal national cohort study in South Korea
Source: Front Psychiatry. 2022 Sep 6;13:607892. doi: 10.3389/fpsyt.2022.607892 (PMC9486390; doi:10.3389/fpsyt.2022.607892)
Supplement: Supplementary file 1 [file Data_Sheet_1.docx]

**Supplementary Table 1**

Demographic and socioeconomic characteristics of the study population

|  | | **Total suicide attempters**  **(N = 5,874)** | **Psychiatric service use**  **prior to the index suicide attempt^a^** | | |
| --- | --- | --- | --- | --- | --- |
|  |  |  | **Yes**  **(N = 1,798 )** | **No**  **(N = 4,076)** | ***p*-value** |
|  |  | **N (%)** | **N (%)** | **N (%)** |  |
| Sex | |  |  |  | <0.001 |
|  | Male | 3,027 (51.5) | 824 (45.8) | 2,203 (54.1) |  |
|  | Female | 2,847 (48.5) | 974 (54.2) | 1,873 (46.0) |  |
| Age (years) | |  |  |  | 0.037 |
|  | 10–19 | 341 (5.8) | 93 (5.2) | 248 (6.1) |  |
|  | 20–29 | 711 (12.1) | 215 (12.0) | 496 (12.2) |  |
|  | 30–39 | 910 (15.5) | 301 (16.7) | 609 (14.9) |  |
|  | 40–49 | 1,121 (19.1) | 357 (19.9) | 764 (18.7) |  |
|  | 50–59 | 1,081 (18.4) | 338 (18.8) | 743 (18.2) |  |
|  | 60–69 | 658 (11.2) | 186 (10.3) | 472 (11.6) |  |
|  | 70–79 | 631 (10.7) | 204 (11.4) | 427 (10.5) |  |
|  | ≥80 | 421 (7.2) | 104 (5.8) | 317 (7.8) |  |
| Age group (years) | |  |  |  | 0.152 |
|  | ≤18 | 268 (4.6) | 69 (3.8) | 199 (4.9) |  |
|  | 19–34 | 1,201 (20.5) | 372 (20.7) | 829 (20.3) |  |
|  | 35–49 | 1,614 (27.5) | 525 (29.2) | 1,089 (26.7) |  |
|  | 50–64 | 1,434 (24.4) | 429 (23.9) | 1,005 (24.7) |  |
|  | ≥65 | 1,357 (23.1) | 403 (22.4) | 954 (23.4) |  |
| Residential area | |  |  |  | 0.032 |
|  | Seoul | 1,295 (22.1) | 394 (21.9) | 901 (22.1) |  |
|  | Busan | 144 (2.5) | 50 (2.8) | 94 (2.3) |  |
|  | Daegu | 86 (1.5) | 29 (1.6) | 57 (1.4) |  |
|  | Incheon | 341 (5.8) | 85 (4.7) | 256 (6.3) |  |
|  | Gwangju | 177 (3.0) | 48 (2.7) | 129 (3.2) |  |
|  | Daejeon | 200 (3.4) | 78 (4.3) | 122 (3.0) |  |
|  | Ulsan | 57 (1.0) | 17 (1.0) | 40 (1.0) |  |
|  | Sejong | 18 (0.3) | 5 (0.3) | 13 (0.3) |  |
|  | Gyeonggi | 1,341 (22.8) | 436 (24.3) | 905 (22.2) |  |
|  | Gangwon | 204 (3.5) | 63 (3.5) | 141 (3.5) |  |
|  | Chungbuk | 234 (4.0) | 78 (4.3) | 156 (3.8) |  |
|  | Chungnam | 489 (8.3) | 130 (7.2) | 359 (8.8) |  |
|  | Jeonbuk | 414 (7.1) | 122 (6.8) | 292 (7.2) |  |
|  | Jeonnam | 333 (5.7) | 88 (4.9) | 245 (6.0) |  |
|  | Gyeongbuk | 263 (4.5) | 81 (4.5) | 182 (4.5) |  |
|  | Gyeongnam | 230 (3.9) | 75 (4.2) | 155 (3.8) |  |
|  | Jeju | 44 (0.8) | 19 (1.1) | 25 (0.6) |  |
| Income class^b^ | |  |  |  | <0.001 |
|  | Lower | 1,407 (27.4) | 381 (26.3) | 1,026 (27.8) |  |
|  | Middle | 1,812 (35.2) | 469 (32.3) | 1,343 (36.4) |  |
|  | Upper | 1,924 (37.4) | 601 (41.4) | 1,323 (35.8) |  |
| Disability type | |  |  |  | <0.001 |
|  | None | 5,055 (86.1) | 1,482 (82.4) | 3573 (87.7) |  |
|  | Psychiatric disability | 75 (1.3) | 75 (4.2) | 0 (0) |  |
|  | Physical disability | 106 (1.8) | 28 (1.6) | 78 (1.9) |  |
|  | Brain lesion disability | 63 (1.1) | 22 (1.2) | 41 (1.0) |  |
|  | Visual disability | 81 (1.4) | 21 (1.2) | 60 (1.5) |  |
|  | Hearing impairment | 75 (1.3) | 75 (4.2) | 0 (0) |  |
|  | Other disabilities^c^ | 96 (1.6) | 47 (2.6) | 49 (1.2) |  |
| Disability severity | |  |  |  | <0.001 |
|  | No disability | 5,055 (86.1) | 1,482 (82.4) | 3,573 (87.7) |  |
|  | Mild | 460 (7.8) | 147 (8.2) | 313 (7.7) |  |
|  | Severe | 359 (6.1) | 169 (9.4) | 190 (4.7) |  |

^a^Psychiatric service use within six months before the index suicide attempt

^b^Income class stratified according to level of health insurance payment; lower class

^c^Includes speech impairment and intellectual disabili

**Supplementary Table 2**

Method used for the index suicide attempt

|  | | **Suicide attempters**  **(N = 5,874 )** | **Psychiatric service use**  **prior to the index suicide attempt^a^** | | |
| --- | --- | --- | --- | --- | --- |
|  |  |  | **Yes**  **(N = 1,798 )** | **No**  **(N = 4,076)** | ***p*-value** |
|  |  | **N (%)** | **N (%)** | **N (%)** |  |
| Poisoning | |  |  |  | <0.001 |
|  | Antiepileptic, sedative-hypnotic, anti-parkinsonism, and psychotropic drugs (X61) | 1,015 (17.3) | 474 (26.4) | 541 (30.1) |  |
|  | Gases and vapors (X67) | 199 (3.4) | 54 (3.0) | 145 (8.1) |  |
|  | Pesticides (X68) | 930 (15.8) | 215 (12.0) | 715 (39.8) |  |
|  | Other poisoning (X60, 62, 63, 64, 65, 66, 69) | 1,905 (32.4) | 431 (24.0) | 1474 (82.0) |  |
| Hanging, strangulation, and suffocation (X70) | | 702 (12.0) | 202 (11.2) | 500 (12.3) |  |
| Drowning and submersion (X71) | | 37 (0.6) | 10 (0.6) | 27 (0.7) |  |
| Firearm discharge (X72, 73, 74) | | 13 (0.2) | 1 (0.1) | 12 (0.3) |  |
| Explosive material (X75) | | 5 (0.1) | 0 (0) | 5 (0.1) |  |
| Smoke, fire, flame, steam, hot vapors, and hot objects (X76, 77) | | 31 (0.5) | 9 (0.5) | 22 (0.5) |  |
| Sharp or blunt object (X78, 79) | | 640 (10.9) | 241 (13.4) | 399 (9.8) |  |
| Jumping (X80, 81) | | 76 (1.3) | 23 (1.3) | 53 (1.3) |  |
| Crashing of motor vehicle (X82) | | 4 (0.1) | 1 (0.1) | 3 (0.1) |  |
| Others (X83, 84) | | 317 (5.4) | 137 (7.6) | 180 (4.4) |  |

^a^Psychiatric service use within six months before the index suicide attempt

**Supplementary Table 3**

Adjusted hazard ratios of potential risk factors for suicide reattempt among suicide attempters who had not reattempted suicide or died within the three-month period after the index suicide attempt (n = 4,842)^a^

|  | | **HR (95% CI)** | ***p*-value** | **Psychiatric service use**  **prior to the index suicide attempt^b^** | | | |
| --- | --- | --- | --- | --- | --- | --- | --- |
|  |  |  |  | **Yes**  **(N = 1,480 )** | | **No (N = 3,362)** | |
|  |  |  |  | **HR (95% CI)** | ***p*** | **HR (95% CI)** | ***p*** |
| Female sex | | 0.86 (0.60–1.24) | 0.418 | 0.95 (0.56–1.60) | 0.835 | 0.74 (0.44–1.24) | 0.251 |
| Age (years) | |  |  |  |  |  |  |
|  | ≤18 | 1 (Ref.) |  | 1 (Ref.) |  | 1 (Ref.) |  |
|  | 19–34 | 0.81 (0.33–1.98) | 0.642 | 0.58 (0.18–1.86) | 0.357 | 1.29 (0.29–5.80) | 0.740 |
|  | 35–49 | 0.84 (0.35–2.02) | 0.697 | 0.47 (0.15–1.51) | 0.207 | 1.58 (0.36–6.85) | 0.543 |
|  | 50–64 | 0.91 (0.37–2.21) | 0.826 | 0.53 (0.16–1.76) | 0.300 | 1.65 (0.38–7.27) | 0.506 |
|  | ≥65 | 0.59 (0.23–1.54) | 0.283 | 0.34 (0.09–1.24) | 0.101 | 1.16 (0.25–5.46) | 0.855 |
| Disability severity | |  |  |  |  |  |  |
|  | No disability | 1 (Ref.) |  | 1 (Ref.) |  | 1 (Ref.) |  |
|  | Severe | 1.74 (0.95–3.20) | 0.074 | 1.97 (0.91–4.26) | 0.086 | 1.40 (0.49–3.99) | 0.533 |
|  | Mild | 2.14 (1.24–3.70) | 0.006 | 2.95 (1.40–6.20) | 0.004 | 1.55 (0.68–3.56) | 0.299 |
| Psychiatric diagnosis^c^ | |  |  |  |  |  |  |
|  | Adjustment disorder | 0.66 (0.24–1.79) | 0.410 | 0.69 (0.21–2.26) | 0.539 | 0.56 (0.08–4.08) | 0.563 |
|  | Depressive disorder | 0.97 (0.63–1.50) | 0.897 | 0.95 (0.53–1.68) | 0.849 | 0.81 (0.39–1.67) | 0.572 |
|  | Schizophrenia spectrum disorder | 1.07 (0.57–2.01) | 0.843 | 0.96 (0.47–1.96) | 0.918 | 0.88 (0.18–4.19) | 0.867 |
|  | Bipolar disorder | 1.11 (0.61–2.01) | 0.742 | 1.03 (0.53–2.00) | 0.924 | 0.98 (0.22–4.46) | 0.982 |
|  | Insomnia | 1.50 (0.97–2.34) | 0.071 | 1.16 (0.55–2.44) | 0.700 | 1.60 (0.91–2.81) | 0.101 |
|  | Other psychiatric disorders | 1.48 (1.00–2.20) | 0.052 | 1.38 (0.78–2.44) | 0.274 | 1.32 (0.75–2.33) | 0.342 |
| Frequency of visits to a psychiatrist within three months after the index suicide attempt | |  |  |  |  |  |  |
|  | 0 | 1 (Ref.) |  | 1 (Ref.) |  | 1 (Ref.) |  |
|  | 1–2 | 0.25 (0.10–0.59) | 0.002 | 0.34 (0.11–1.02) | 0.054 | 0.08 (0.01–0.57) | 0.012 |
|  | ≥3 | 1.08 (0.57–2.05) | 0.822 | 0.84 (0.35–2.03) | 0.699 | 0.97 (0.32–2.95) | 0.952 |
|  | Admission to psychiatry | 0.79 (0.41–1.55) | 0.497 | 0.62 (0.25–1.56) | 0.313 | 0.75 (0.24–2.33) | 0.616 |
| Psychiatric medication use within 3 months | |  |  |  |  |  |  |
|  | No psychiatric medication | 1 (Ref.) |  | 1 (Ref.) |  | 1 (Ref.) |  |
|  | Antidepressants | 1.49 (0.92–2.43) | 0.105 | 1.47 (0.82–2.66) | 0.197 | 1.48 (0.63–3.45) | 0.365 |
|  | Antipsychotics | 1.28 (0.74–2.21) | 0.372 | 1.07 (0.57–2.02) | 0.824 | 1.76 (0.62–4.97) | 0.290 |
|  | Benzodiazepines | 0.99 (0.63–1.55) | 0.964 | 0.80 (0.43–1.50) | 0.486 | 1.08 (0.58–2.03) | 0.804 |
|  | Stimulants | 0.54 (0.08–3.91) | 0.545 | 0.97 (0.13–7.16) | 0.975 | 0 (0) | 0.980 |
|  | Mood stabilizers | 1.18 (0.67–2.10) | 0.566 | 1.24 (0.65–2.34) | 0.514 | 0.68 (0.15–3.12) | 0.622 |
|  | Zolpidem | 0.58 (0.34–0.98) | 0.042 | 0.75 (0.41–1.36) | 0.335 | 0.22 (0.05–0.95) | 0.042 |

Abbreviations: HR, hazard ratio; CI, confidence interval

^a^Adjusted for sex, age, disability severity, psychiatric diagnosis, frequency of visits to a psychiatrist during 3 months, and psychiatric medication use within three months after the index suicide attempt

^b^Psychiatric service use within six months before the index suicide attempt

^c^Insomnia was diagnosed by doctors of any department and the others were diagnosed by a psychiatrist (as the main diagnosis) during the study period
